# Physical Fitness and Motor Competence in Upper Austrian Elementary School Children—Study Protocol and Preliminary Findings of a State-Wide Fitness Testing Program

**DOI:** 10.3389/fspor.2021.635478

**Published:** 2021-02-22

**Authors:** Clemens Drenowatz, Franz Hinterkörner, Klaus Greier

**Affiliations:** ^1^Division of Sport, Physical Activity and Health, University of Education Upper Austria, Linz, Austria; ^2^Olympic Training Center Upper Austria, Linz, Austria; ^3^Division of Physical Education, Private Educational College (Kirchliche Pädagogische Hochschule - Edith Stein), Stams, Austria; ^4^Department of Sports Science, Leopold-Franzens University Innsbruck, Innsbruck, Austria

**Keywords:** cardiorespiratory endurance, muscular strength, motor skills, motor competence, body weight, youth

## Abstract

Motor competence and physical fitness are key components for the promotion of an active and healthy lifestyle. Poor motor competence and low physical fitness in children, therefore, are a major threat to future public health. Even though the assessment of physical fitness and motor competence *per se* does not enhance these entities, fitness tests can provide important information for intervention strategies. Fitness tests may also motivate children to become more active in order to increase their physical abilities. In the school-year 2016/17 the Upper Austrian government initiated the state-wide testing program “wie fit bist du” (how fit are you) in elementary schools, that examined cardiorespiratory fitness, muscular power, speed, agility, flexibility and object control skills along with the assessment of height and weight. Since the beginning of the program more than 18,000 children between 6 and 11 years of age participated in the school-based tests. The results show a significant increase in the prevalence of overweight/obesity with increasing age (*p* > 0.01). Overweight/obese children displayed lower motor competence and physical fitness, except for upper body strength. Further, the improvement in test performance with increasing age was less pronounced in overweight/obese children compared to their normal weight peers. In fact, distance covered during the 6-min run did not improve throughout the elementary school years in overweight/obese children. Given the importance of motor competence and physical fitness for general development and well-being, physical education should be considered a viable setting for the promotion of these entities as a majority of children can be reached independent of their socio-economic background. In order to provide adequate movement experiences that enhance motor competence and physical fitness while ensuring a motivating environment, objective information on current ability levels are required. The implementation of fitness monitoring at young ages, therefore, can be an important contributor for the promotion of an active and healthy lifestyle.

## Introduction

Physical fitness and motor competence are considered key components for the development and general health in children and adolescents (Dwyer et al., 2009; Ruiz et al., 2009; Robinson et al., 2015; Hamer et al., 2020; Raghuveer et al., 2020). There is considerable evidence for beneficial associations of physical fitness with body weight (Ortega et al., 2008; Rauner et al., 2013), chronic disease risk (Ortega et al., 2008; Ruiz et al., 2009; Grøntved et al., 2015; Lang et al., 2018), cognitive development and academic performance (Ortega et al., 2008; Santana et al., 2017; Marques et al., 2018; Mintjens et al., 2018) as well as mental health (Ortega et al., 2008; Lubans et al., 2016). Similarly, motor competence has been associated with body weight (Lopes et al., 2012; Robinson et al., 2015; Barnett et al., 2016) in addition to self-efficacy and general well-being (Robinson et al., 2015). Further, motor competence is directly associated with physical fitness as it reflects the ability to perform goal-directed movements that involve large muscle groups or the whole body (Robinson et al., 2015; Barnett et al., 2016). Accordingly, motor competence provides the foundation for various sport-specific skills, particularly during middle and late childhood (Clark and Metcalf, 2002; Stodden et al., 2008), which will influence physical fitness (Robinson et al., 2015; Barnett et al., 2016) and subsequent physical activity (PA) (Barnett et al., 2009; Lopes et al., 2011; Lloyd et al., 2014). Physical fitness is also an important component in the promotion of active leisure time choices as it has been defined as the ability to complete daily activities without undue fatigue that provides sufficient energy reserves to engage in active recreational pursuits (Malina and Katzmarzyk, 2006). In addition, high motor competence and physical fitness have been suggested to induce relatively permanent behavioral choices that transfer into adulthood (Barnett et al., 2009; Lai et al., 2014; Telama et al., 2014; Robinson et al., 2015; García-Hermoso et al., 2019) and are considered key components in the promotion of an active lifestyle (Stodden et al., 2008). Nevertheless, physical fitness and motor competence have declined in children and adolescents over the last several decades (Moraes Ferrari et al., 2013; Brian et al., 2018; Tomkinson et al., 2019). Available data indicates “below normal” muscular fitness in 74% of Czech children (Müllerova et al., 2015) and less than half of US adolescents are believed to achieve healthy fitness levels (Gahche et al., 2014). At the same time the prevalence of overweight and obesity in children increased, which also has significant impact on current and future health (Llewellyn et al., 2016; NCD Risk Factor Collaboration, 2017). Given these trends in body weight and physical fitness, additional efforts to promote an active and healthy lifestyle are needed.

The elementary school years appear to be a particularly critical period for the development of physical fitness and motor competence (Augste and Jaitner, 2010). Motor competence and physical fitness, however, do not develop naturally (Robinson et al., 2015); rather, motor development is described as a learning process that is driven by structural and functional changes of the body and the environment (Clark, 1995), which requires nurturing experiences (Robinson et al., 2015). Changes in the social and built environment, however, have led to a decline in PA at young ages, which hinders motor development (Dordel, 2000; Sygusch, 2006; Bös et al., 2008). The detrimental association of insufficient PA, low physical fitness, poor motor competence and associated health outcomes is also referred to as pediatric inactivity triad, which is a considerable risk for future public health (Faigenbaum et al., 2018). In order to reach a large number of children, independent of their socio-economic background, schools are a prime setting for the promotion of PA and health in children and adolescents (Peralta et al., 2020). There is also evidence on beneficial effects of structured school-based interventions on physical fitness and motor competence (García-Hermoso et al., 2020).

Even though the assessment of motor competence and physical fitness does not improve these entities *per se*, it does provide information on current abilities, which is crucial for the selection of adequate movement experiences that facilitate motor development. Further, repeated assessments provide valuable insights into the efficacy of various intervention strategies and potentially serve as motivation to engage in activities that promote motor competence and physical fitness (Wiersma and Sherman, 2008; Jaakkola et al., 2013). Harris and Cale (2019), therefore, argue that appropriately performed and well-incorporated fitness monitoring should be part of the curriculum due to its viable role in supporting an active and healthy lifestyle. The German Olympic Sports Association also recommends regular assessments of motor competence and physical fitness starting from the elementary school years beyond its use for talent identification (Deutscher Olympischer Sportbund, 2013). Given these recommendations the Upper Austrian government initiated the project “wie fit bist du?” (how fit are you?), which provides a large-scale assessment of motor competence and physical fitness in Upper Austrian elementary school children. The purpose of this article is to provide a description of the study protocol and individual fitness assessments. Further, preliminary cross-sectional results addressing differences in physical fitness by age and weight status are shown.

## Materials and Methods

The project started in the school-year 2016/17. All elementary schools in the federal state of Upper Austria were informed about the project. During the first year of data collection only elementary students in grades 2 and 3 were eligible for participation. In subsequent years, grade 4 students were included as part of follow-up measurements and first grade students were included as this was requested by participating schools. Study procedures have been approved by the Upper Austrian School Board and are in accordance with the 2008 declaration of Helsinki. Written informed consent was obtained from participating schools (school board and classroom teacher) and parents. Children provided oral assent at the time of measurement.

### Test Items

#### Anthropometric Measurements

Anthropometric measurements were taken according to standard procedures with children in gym clothes and barefoot. A portable stadiometer (SECA 2013, Seca, Hamburg, Germany) was used to measure body height to the nearest 0.5 cm. Body weight was measured with an electronic scale (Seca 878 dr, Seca, Hamburg, Germany) to the nearest 0.1 kg. Subsequently body max index (BMI) was calculated (kg/m^2^) and converted to BMI percentiles (BMIPCT) using German reference values (Kromeyer-Hauschild et al., 2001). Children with a BMIPCT above the 90th percentile were classified as overweight and children above the 97th percentile were classified as obese. Children with a BMIPCT of <10 were considered underweight.

The participants completed a total of eight tests that assessed various components of physical fitness (cardiorespiratory endurance, muscular strength, muscular power, speed, and flexibility) as well as motor competence (agility and object control).

#### Cardiorespiratory Endurance

Distance covered during a 6-min run was used as indicator for cardiorespiratory endurance. The running track was marked in the school's gymnasium with four cones that were set up 2.5 m from each corner of the gymnasium. The distance of one lap was measured with a measuring wheel in duplicate to assure accuracy. Total distance covered was determined to the nearest meter [Distance (m) = lap (m) × number of laps + distance of last partial lap]. In order avoid congestion during the run no more than 4 participants were starting in each corner per trial. Prior to the beginning of the test participants were instructed to complete as many laps as possible within 6 min—in case of fatigue participants were told to continue walking rather than maintain still. Laps were counted by trained technicians. Participants received verbal time warnings at minutes 3, 4, and 5, as well as 30 s prior to the end. The last 10 s were counted down and participants were asked to remain at their position at the end of the 6 min interval in order to determine the distance covered during the final lap.

#### Muscular Strength and Power

Muscular strength was assessed via a medicine ball push (1 kg). The participant was asked to hold the medicine ball with two hands in front of the chest and toss the ball for maximum distance using only arms and upper body, while keeping both legs extended and feet on the ground. Distance was recorded to the nearest 10 cm using a measurement tape. Overhead throwing motions or stepping over the 0 cm line were not allowed to complete this task.

In addition, a counter movement jump was used as indicator of lower extremity power. The counter movement jump was performed on a contact measuring plate (TDS Linz, Austria) with the participant standing with hands at the waist. Upon the command “and jump” the participant jumped for maximal vertical height while keeping the hands at the waist. Legs were supposed to stay extended during the flight phase in order to avoid landing in a tucked position as this would affect the measurement.

#### Speed

Linear speed was assessed via a 10 m sprint. Participants started in an up-right position 1 m behind the starting line after receiving a ready-signal from the technician. In order to avoid a deceleration prior to the finish line, a finishing area was marked 3 m after the actual 10 m mark. Running time to cover the 10 m distance was measured via photocell measurement (TDS Linz, Austria).

In addition, a tapping test was performed, in which the participant was asked to perform as many alternating contacts with the left and right foot as possible within 6 s on a contact measuring plate (TDS Linz, Austria).

#### Agility

**Figure 1:**
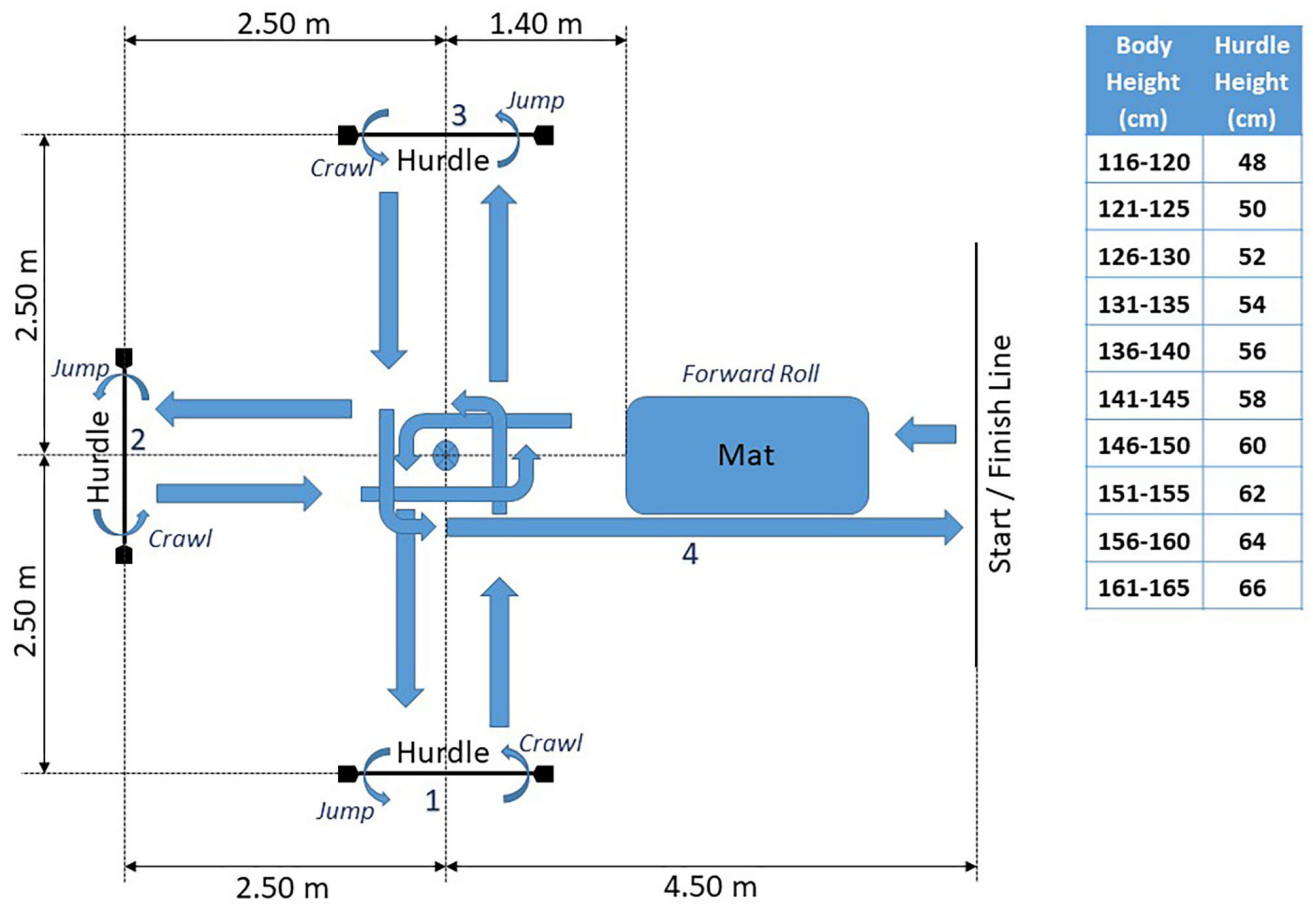
Obstacle run course setup with height chart for hurdles.

Agility was assessed via a standardized obstacle run that includes a forward roll, jumping over as well as moving under hurdles along with directional changes (Figure 1). After crossing the starting line, the participant performed a forward roll on a mat, followed by a 90° turn at the middle pole. Next, the participant jumped over the hurdle and returned by crawling underneath the same hurdle back to the middle pole, where another 90° turn was made. The same procedure was repeated for the next two hurdles. After completing all three hurdles the participant ran back to the starting line (no second forward roll). The height of the hurdles was set based on the participant's body height and completion time was measured by hand to the nearest 1/100 s. In case a participant was unable to perform a forward roll 5 s were added to the time measured.

#### Flexibility

Flexibility was assessed via a stand-and-reach test. The participant stood barefoot on the measurement box with the knees fully extended and legs being closed. Then, the participant was asked to reach as far as possible toward or beyond the toes along the measurement scale by bending at the hip while keeping the knees extended. At maximum extension the position needed to be held for 2 s to measure distance from the toes in mm. Positive values indicate reaching beyond the toes, while negative values indicate not reaching the toes.

#### Object Control

Ball handling skills were assessed by a 30-s throw-and-catch task with a European handball (size 1). The participant was instructed to throw the ball (using a one-handed overhead motion) from 1.5 m distance against a wall and catch the returning ball with two hands prior to it touching the ground. This pattern was repeated over 30 s in an attempt to complete as many throws and catches as possible. Four extra balls were prepared next to the participant in case a ball bounced away. Only successful catches following a proper one-handed overhead throw were counted. Attempts when the ball was not caught, when the ball bounced on the ground or the participant stepped over the 1.5 m line were disregarded.

### Testing Procedures

All assessments were taken by trained technicians in the participating school's gymnasium within 90–120 min. Every school visit followed the procedures detailed in a written manual that included information on set up of the fitness assessments, welcoming students, warm up, test administration and reporting of test results. In addition, a senior staff of the project was present to supervise procedures. All children of the respective class, who were able to participate in PE the day of the fitness tests participated in the assessments but only data of children with parental consent were entered on site in a specifically developed software. There were no make-up days for children who were sick and missed school that day or for children who were not able to participate in the fitness tests. After measuring height (kg) and weight (cm), participants completed a 5-min standardized warm up. Subsequently, the fitness tests were completed in random order, except for the 6-min run, which was completed at the end of the testing session in order to avoid undue fatigue. All tests were verbally explained and shown to the participants prior to the respective assessments. Participants performed each test twice, with sufficient recovery time between trials, except for the vertical jump (three trials back-to-back) and the 6-min run (one trial). The best attempts were used for further analyses.

### Statistical Analyses

Participants were stratified by chronological age into five age groups (6.0–6.9; 7.0–7.9; 8.0–8.9; 9.0–9.9; 10.0–10.9). Descriptive statistics were calculated and data were checked for normal distribution. Chi square tests were used to examine differences in categorical variables. Multivariate analysis of variance (MANOVA) was used to examine differences across the fitness tests by sex and age group. In addition, differences in physical fitness and motor competence between overweight/obese and normal weight participants by age group were examined via 2 × 5 MANOVA. All statistical analyses were performed with SPSS 26.0 with a significance level of *p* < 0.05 and Bonferroni adjustment for multiple comparisons.

## Results

Since the start of the project in October 2016 a total of 28,481 assessments were completed until July 2019. During that time span 18,746 children (51% male) between the ages 6 and 11 years were tested at least once. A total of 18,168 children had valid data for all measurements and were included in the analyses. There were no significant differences in sex distribution and age between children with incomplete data and those included in the analyses. An overview of anthropometric characteristics and sex distribution by age group is provided in Table 1.

**Table 1:** Anthropometric characteristics by age group.

**Age group (*N*, % male)**	**Age(years)**	**Height (cm)**	**Weight (kg)**	**BMI Percentile**
6–7 years (352, 56.0%)	6.7 ± 0.2	122.7 ± 5.3	24.3 ± 4.6	51.3 ± 29.8
7–8 years (5,875, 49.0%)	7.7 ± 0.2	128.6 ± 5.7	27.2 ± 5.4	50.8 ± 28.7
8–9 years (8,156, 51.2%)	8.4 ± 0.3	132.5 ± 5.9	29.8 ± 6.6	51.6 ± 29.7
9–10 years (3,144, 55.2%)	9.4 ± 0.3	137.4 ± 6.5	33.6 ± 8.2	53.9 ± 31.2
10–11 years (641, 52.7%)	10.4 ± 0.3	142.1 ± 6.6	37.1 ± 9.3	53.8 ± 31.8

*Values are mean ± SD*.

Across the entire sample 8.4% were considered overweight and 6.3% of the children were considered obese. More boys than girls were overweight and obese (8.7 vs. 8.1% and 6.6 vs. 5.9%, respectively). The prevalence of underweight was 8.5% with a higher prevalence in boys compared to girls (9.6 vs. 7.4%). There was also an increase in the prevalence of overweight and obesity across age groups from 7.1 and 6.3% in 6–7 year-old children to 11.2% and 7.6% in 10–11 year-old children, respectively. The prevalence of underweight remained relatively stable (9.7% in 6–7 year-old children vs. 10.1% in 10–11 year-old children).

Despite the rising prevalence of overweight/obesity with increasing age physical fitness and motor competence improved significantly across age groups (*p* for trend < 0.01), except for flexibility, where a significant decline was observed with increasing age (*p* for trend < 0.01). Boys performed better than girls at all test items, except for the stand and reach test, which was better in girls compared to boys (*p* < 0.01, Table 2).

**Table 2:** Physical fitness and motor competence by age group and sex.

		**6–7 Years**	**7–8 Years**	**8–9 Years**	**9–10 Years**	**10–11 Years**
6-min Run (m)	Total	921 ± 122	970 ± 126	987 ± 133	994 ± 144	1,005 ± 150
	Boys	933 ± 127	1,006 ± 127	1,021 ± 136	1,019 ± 154	1,026 ± 155
	Girls	906 ± 115	936 ± 116	951 ± 120	965 ± 123	982 ± 142
Ball push (m)	Total	2.7 ± 0.5	3.2 ± 0.7	3.5 ± 0.7	4.0 ± 0.8	4.4 ± 0.8
	Boys	2.8 ± 0.6	3.4 ± 0.6	3.8 ± 0.6	4.2 ± 0.8	4.6 ± 0.8
	Girls	2.5 ± 0.4	3.0 ± 0.5	3.3 ± 0.6	3.7 ± 0.7	4.1 ± 0.7
CMJ (cm)	Total	17.4 ± 3.2	19.2 ± 3.4	20.0 ± 3.8	20.7 ± 4.2	21.7 ± 4.6
	Boys	17.4 ± 3.3	19.7 ± 3.5	20.5 ± 3.9	21.2 ± 4.3	22.1 ± 4.6
	Girls	17.4 ± 3.2	18.8 ± 3.3	19.5 ± 3.6	20.1 ± 4.0	21.2 ± 4.6
10 m Sprint (s)	Total	2.4 ± 0.2	2.3 ± 0.2	2.3 ± 0.2	2.2 ± 0.2	2.2 ± 0.2
	Boys	2.4 ± 0.2	2.3 ± 0.2	2.2 ± 0.2	2.2 ± 0.2	2.2 ± 0.2
	Girls	2.4 ± 0.2	2.3 ± 0.2	2.3 ± 0.2	2.3 ± 0.2	2.2 ± 0.2
Tapping (# in 6 s)	Total	39.1 ± 7.2	43.1 ± 6.8	45.3 ± 7.3	47.5 ± 7.8	49.5 ± 8.6
	Boys	40.4 ± 7.0	45.1 ± 6.5	47.0 ± 6.9	49.2 ± 7.4	50.8 ± 8.7
	Girls	37.5 ± 7.1	41.1 ± 6.6	43.4 ± 7.2	45.5 ± 7.9	48.0 ± 8.4
Obstacle run (s)	Total	22.7 ± 3.8	20.5 ± 3.3	19.9 ± 3.9	19.3 ± 3.9	18.8 ± 4.2
	Boys	22.7 ± 4.1	19.9 ± 3.3	19.4 ± 3.6	18.9 ± 4.0	18.3 ± 3.8
	Girls	22.8 ± 3.5	21.0 ± 3.2	20.3 ± 3.5	19.8 ± 3.7	19.4 ± 4.6
Stand and reach (cm)	Total	2.4 ± 6.0	2.3 ± 6.2	1.7 ± 6.6	0.6 ± 7.2	0.3 ± 7.2
	Boys	1.4 ± 5.4	0.8 ± 5.9	0.1 ± 6.4	−1.0 ± 7.0	−1.5 ± 7.3
	Girls	3.6 ± 6.4	3.7 ± 6.2	3.4 ± 6.5	2.5 ± 7.0	2.4 ± 6.5
Throw and catch (# in 30 s)	Total	5.6 ± 5.6	11.7 ± 6.9	15.7 ± 6.9	19.9 ± 6.8	22.9 ± 6.9
	Boys	7.0 ± 6.0	14.2 ± 6.7	17.8 ± 6.7	21.3 ± 6.6	24.1 ± 7.1
	Girls	3.9 ± 4.6	9.3 ± 6.2	13.6 ± 6.5	18.1 ± 6.6	21.7 ± 6.3

*Values are mean ± SD*.

*CMJ, counter movement jump*.

Across the entire sample, there were significant differences in physical fitness between normal weight and overweight/obese participants (*p* < 0.01), except for the throw and catch task. Normal weight participants displayed better performance than their overweight/obese peers at the 6-min run, 10 m sprint, counter movement jump, tapping, obstacle run and stand and reach test. Ball push performance, on the other hand, was better in overweight/obese children compared to their normal weight peers.

**Figure 2:**
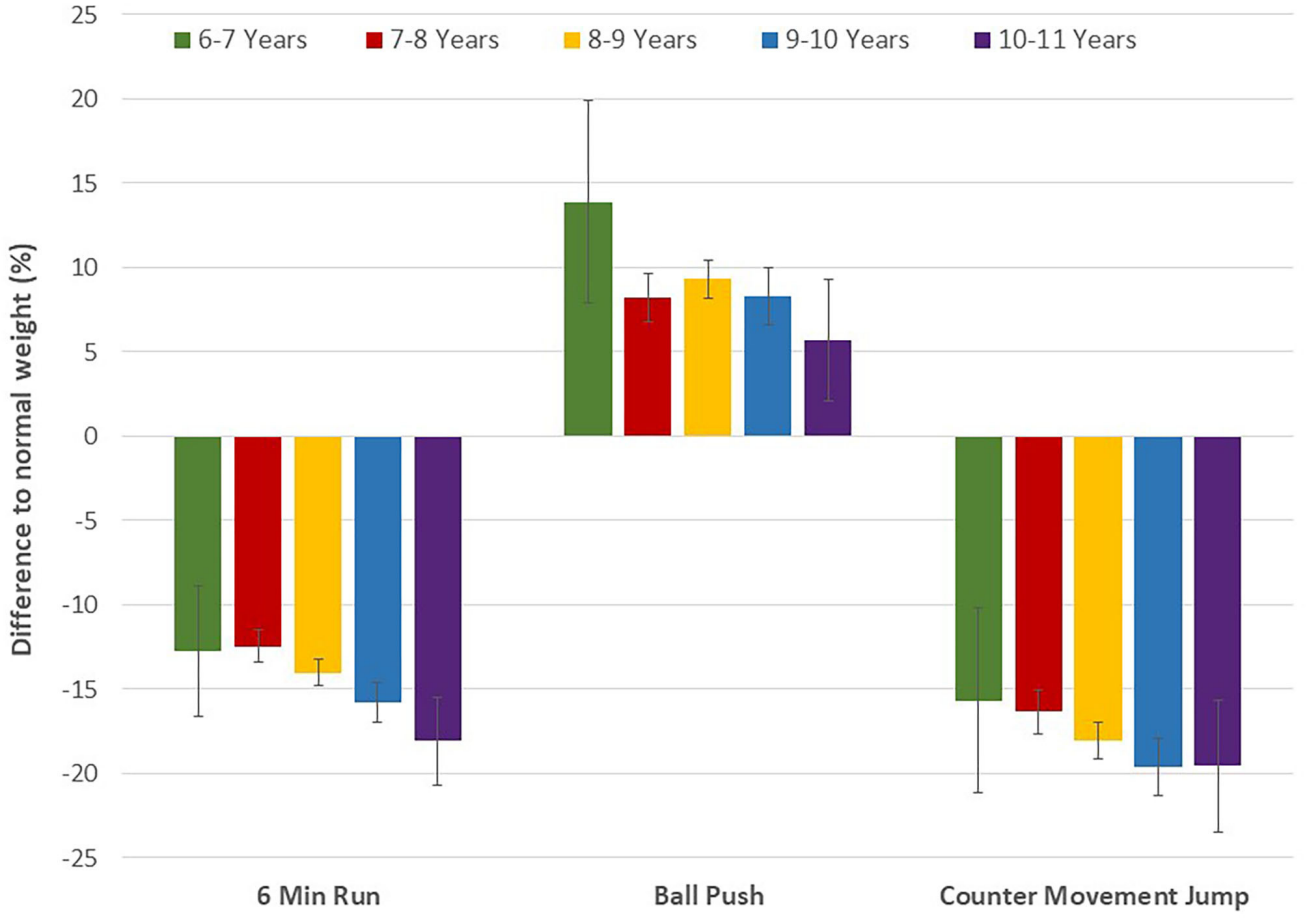
Differences in endurance, strength, and power between normal weight and overweight/obese children by age group (from ages 6–7 to 10–11 left to right). Values are means with 95% CI.

**Figure 3:**
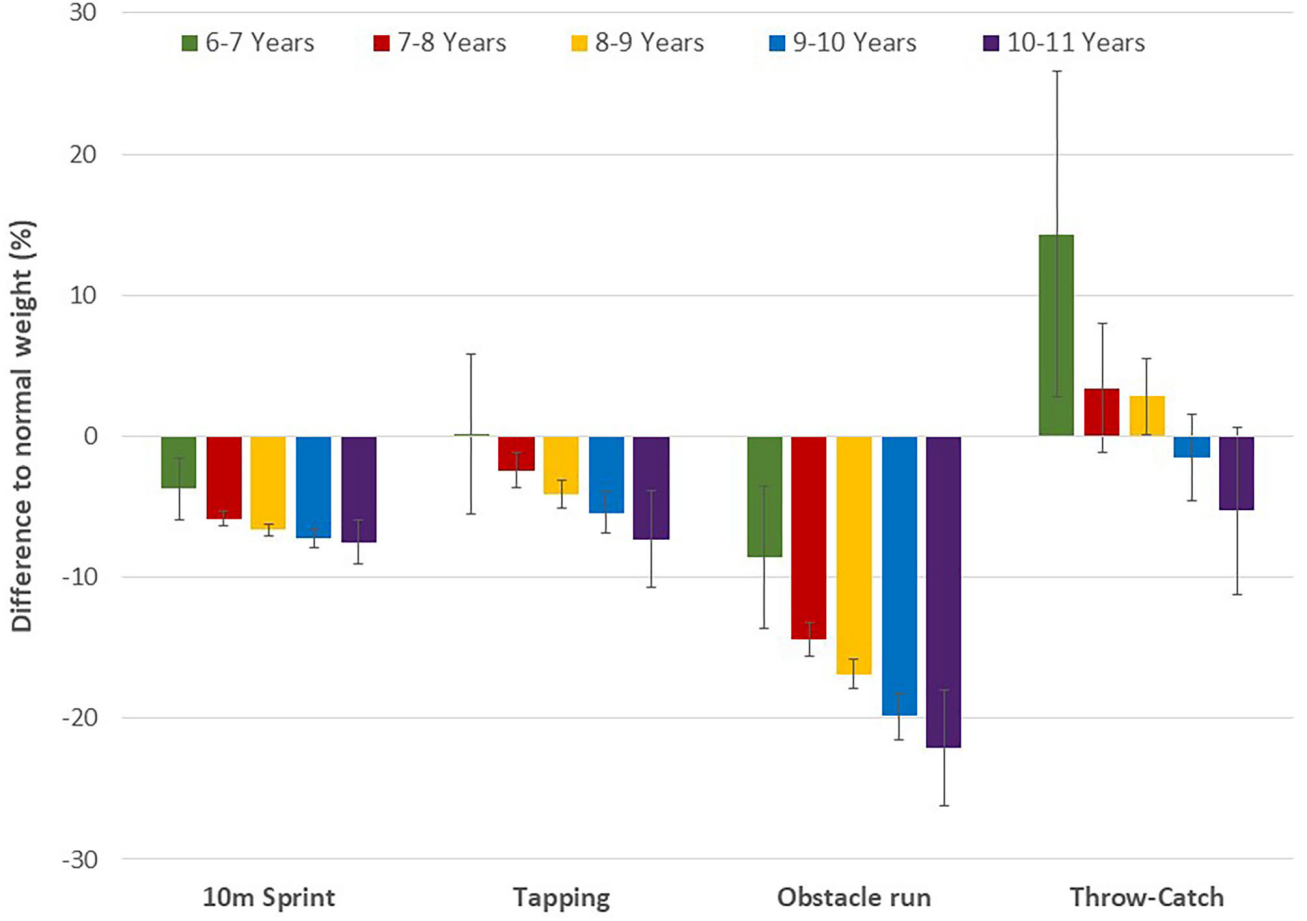
Differences in speed, agility, and object control between normal weight and overweight/obese children by age group (from ages 6–7 to 10–11 left to right). Values are means with 95% CI.

There was a significant weight category by age group interaction on physical fitness (Wilks' Lambda = 0.99, *p* < 0.01). Interaction effects of the individual fitness tests were significant for all tests, except for the stand-and-reach test. Specifically, the difference between normal weight and overweight/obese children became more pronounced for the 6-min run, counter movement jump, 10 m sprint, tapping and obstacle run (*p* < 0.01), while the performance difference declined for the medicine ball push (*p* < 0.01) (Figures 2, 3). At the throw and catch test overweight/obese children performed better than their peers at the younger ages, while normal weight children displayed better results in the older age group. Except for the stand-and-reach test overweight/obese participants showed smaller performance differences across age groups compared to their normal weight peers (Table 3), which indicates a smaller improvement in physical fitness and motor competence in children with excess body weight. In fact, 6-min run performance did not change across age groups in overweight/obese, while there was a significant improvement with increasing age in normal weight children. These results remained essentially the same after adjusting for sex.

**Table 3:** Physical fitness and motor competence in normal weight and overweight/obese children by age group.

		**6–7 Years**	**7–8 Years**	**8–9 Years**	**9–10 Years**	**10–11 Years**
6-min Run (m)	NW	937 ± 107	985 ± 120	1,007 ± 123	1,024 ± 132	1,039 ± 129
	OW/OB	820 ± 163	864 ± 122	869 ± 126	868 ± 122	858 ± 145
Ball push (m)	NW	2.6 ± 0.5	3.2 ± 0.6	3.5 ± 0.6	3.9 ± 0.8	4.3 ± 0.8
	OW/OB	3.0 ± 0.6	3.4 ± 0.6	3.8 ± 0.7	4.2 ± 0.8	4.6 ± 0.8
CMJ (cm)	NW	17.7 ± 3.1	19.6 ± 3.3	20.5 ± 3.6	21.5 ± 4.0	22.5 ± 4.5
	OW/OB	15.0 ± 2.8	16.5 ± 3.2	16.9 ± 3.2	17.4 ± 3.4	18.2 ± 3.5
10 m Sprint (s)	NW	2.42 ± 0.17	2.30 ± 0.15	2.25 ± 0.15	2.20 ± 0.15	2.16 ± 0.17
	OW/OB	2.51 ± 0.19	2.43 ± 0.18	2.40 ± 0.19	2.36 ± 0.18	2.33 ± 0.19
Tapping (# in 6 s)	NW	39.1 ± 6.9	43.2 ± 6.8	45.6 ± 7.2	48.0 ± 7.8	50.1 ± 8.7
	OW/OB	39.2 ± 9.1	42.2 ± 6.7	43.7 ± 7.3	45.4 ± 7.4	46.5 ± 8.0
Obstacle run (s)	NW	22.4 ± 3.7	20.1 ± 3.0	19.4 ± 3.1	18.6 ± 3.3	18.0 ± 3.4
	OW/OB	24.4 ± 4.0	23.1 ± 4.3	22.7 ± 4.7	22.4 ± 4.6	22.2 ± 5.6
Stand and reach (cm)	NW	2.4 ± 5.9	2.4 ± 6.1	1.8 ± 6.6	0.8 ± 7.1	0.5 ± 7.3
	OW/OB	2.0 ± 6.6	2.0 ± 6.6	1.2 ± 6.8	−0.5 ± 7.5	−0.2 ± 7.0
Throw and catch (# in 30 s)	NW	5.3 ± 5.4	11.6 ± 6.9	15.7 ± 6.9	19.9 ± 6.8	23.2 ± 6.7
	OW/OB	7.5 ± 6.8	12.0 ± 6.6	16.1 ± 6.9	19.6 ± 6.7	22.0 ± 7.4

*Values are mean ± SD*.

*NW, normal weight; OW/OB, overweight/obese; CMJ, counter movement jump*.

## Discussion

The intention of the project “wie fit bist du?” was to provide objective information on the fitness level and motor competence of elementary school children in Upper Austria. Such information can provide viable information for the implementation of various movement experiences that improve physical fitness and motor competence, particularly during PE. As expected there was an improvement in physical fitness and motor competence with increasing age, except for flexibility. Further, higher fitness and motor competence levels were observed in boys compared to girls, except for flexibility. There were also considerable differences in performance between normal weight and overweight/obese children. Normal weight children performed better in tasks that required moving their own body weight, while overweight/obese children displayed higher absolute upper body strength (i.e., medicine ball push). These results are consistent with those reported in previous studies (Ortega et al., 2013; Cattuzzo et al., 2016; Fiori et al., 2020). It should also be pointed out that improvements in fitness and motor competence with increasing age were less pronounced in children with excess body weight. Of particular concern is the lack of improvement in cardiorespiratory fitness in overweight/obese children throughout the elementary school years, as cardiorespiratory fitness is an important indicator for future health (Ortega et al., 2008; Raghuveer et al., 2020). Accordingly, these children may require additional support in order to ensure optimal motor development.

The results of this study also support previous reports that many children do not achieve their expected motor competence (Mitchell et al., 2013; Belton et al., 2014), even though it is generally assumed that children achieve mastery of basic movement skills during the elementary school years (Gallahue et al., 2012). Given the importance of physical fitness and motor competence in the promotion of an active and healthy lifestyle (Ortega et al., 2008; Mintjens et al., 2018; Britton et al., 2020), schools and particularly PE should be considered a viable setting for the development of motor competence and physical fitness as most children can be reached independent of their socio-economic background. Even though the benefits of PA on various health-related outcomes have been well-documented (U.S. Department of Health Human Services, 2018), simply ensuring high movement time is not sufficient to promote motor competence and physical fitness; rather deliberate practice with quality movement experiences and feedback is required (Robinson and Goodway, 2009; Barnett et al., 2016; Payne and Isaacs, 2017; Schmutz et al., 2020). Adequate information on current abilities, therefore, is necessary in order to provide movement experiences that enhance motor competence and physical fitness. Accordingly, available research has shown that a targeted approach during PE, which ensures sufficient intensity, improves cardiorespiratory fitness in children even in the absence of additional time dedicated toward PE (García-Hermoso et al., 2020; Peralta et al., 2020). Further, PE lessons with fitness infusion have been shown to increase active learning time in students and, therefore, potentially increase total PA (Lonsdale et al., 2013). Given the higher capacity for PA in individuals with higher cardiorespiratory fitness, children may also be more likely to engage in active behaviors during their leisure time (Raghuveer et al., 2020). Accordingly, health-related fitness has been shown to be the strongest predictor of future PA during the transition from primary to secondary school level and higher PA levels due to increased physical fitness can also have a positive effect on motor development (Britton et al., 2020).

Children with excess body weight are particularly at risk for poor motor competence and low physical fitness. This may also be attributed to the fact that most fitness tests rely on weight-bearing activities, which puts overweight/obese children at a disadvantage and results of the present study indicate similar abilities in non-weight bearing activities. In fact, absolute strength was higher in children with excess body weight compared to their peers. Given the fact that actual and perceived motor competence influence the enjoyment of and motivation toward PA (Loprinzi et al., 2015) strength-related activities may provide a viable option for the promotion of an active lifestyle. Muscular fitness has also been associated with various health benefits (Smith et al., 2014), and is an important correlate of future participation in sports and PA (Behringer et al., 2011; Faigenbaum et al., 2015). In addition to the beneficial association between motor competence, physical fitness and PA, these entities are also associated with academic achievement and perceived cognitive competence (Donnelly et al., 2016). Higher motor competence in children has been associated with improved executive functioning as well as higher-order cognitive skills (van der Fels et al., 2015; Bremer and Cairney, 2016) while low motor competence has been associated with greater attentional difficulties in school tasks and lower self-esteem in general, which negatively affects overall quality of life (Rebondo-Tebar et al., 2021).

Some limitations of this study, however, should be considered when interpreting the results. Given the nature of the project, the main emphasis was on the assessment of fitness and motor competence and no additional data on the school environment as well as socio-economic and cultural background of the participants was obtained. There was also no information on health status and health-related behaviors, such as PA or sports participation, which affect physical fitness and motor competence. In addition, schools volunteered to participate in the project rather than being randomly selected. The utilization of multiple tests that assess various components contributing to physical fitness and motor competence along with a stringent test protocol that was implemented consistently across all assessments and the large sample size, on the other hand, should be considered a strength of the study.

The limited published data on physical fitness in Upper Austrian elementary school children emphasizes the need for a large-scale assessment of physical fitness and motor competence. Even though a state-wide testing program does not enhance physical fitness and motor competence by itself, it does provide objective information on children's current ability level. Such information should subsequently be used for the development of adequate movement experiences that stimulate physical fitness and motor development. The identification of children at risk also facilitates early intervention efforts in order to avoid a vicious cycle of low physical fitness, impaired motor development and various health risks later in life. Repeated assessments may further increase the motivation of children for a more active engagement in PE and potentially increase the likelihood to pursue more active leisure choices in order to improve their results in a subsequent test (Wiersma and Sherman, 2008; Jaakkola et al., 2013). The elementary school years appear to be a particularly critical period for the promotion of an active and healthy lifestyle as various lifestyle habits are established during childhood and early life PA habits have been shown to be associated with PA behavior later in life (Nelson et al., 2006; Telama et al., 2014; Rauner et al., 2015; Rovio et al., 2018; Hayes et al., 2019). Accordingly, fitness monitoring should be incorporated in the elementary school curriculum in order to facilitate the promotion of an active and healthy lifestyle.

## Data Availability Statement

The raw data supporting the conclusions of this article will be made available by the authors, without undue reservation.

## Ethics Statement

The studies involving human participants were reviewed and approved by Upper Austrian School Board. Written informed consent to participate in this study was provided by the participants' legal guardian/next of kin.

## Author Contributions

FH conceptualized the study and managed data collection. CD performed the statistical analysis, prepared the tables and figures, and prepared the manuscript with critical input from KG and FH. All authors have read and approved the submitted version of the manuscript.

## Conflict of Interest

The authors declare that the research was conducted in the absence of any commercial or financial relationships that could be construed as a potential conflict of interest.
